# A fresh view of China’s OFDI, its motivations and risks thereto

**DOI:** 10.1007/s43546-022-00398-w

**Published:** 2022-12-29

**Authors:** M. John Foster

**Affiliations:** Kingston Business School, Kingston University, Kingston upon Thames, KT2 7LB UK

**Keywords:** China, FDI, OFDI, Corruption, Decision making

## Abstract

The aims of this paper are: to examine the key reasons for the locations of and the reasons for China’s OFDI as it boomed and became a major OFDI player in the global economy; and, to assess the existence and nature of possible risks to such a boom continuing. The core of the paper, in its second section, is a time-indexed review of findings relating to the first theme noted above. The broad conclusions are that: China’s OFDI has been a great success in the period 2005–2019; its motivations have included all the usual possibilities, such as access to resources, market access, access to and reverse engineering of technology (very broadly construed), and access to cheap labour for manufacturing; but, it has also sought to wield political influence via infrastructural investment, and its increasingly hostile attitudes in geopolitical terms in the past decade may pose consequential threats to a continuation of the success to date.

## Introduction

The story of foreign direct investment (FDI) as it relates to China, since the declaration of the ‘Open Door Policy’ by Deng Xiao Ping in 1978, has two distinct parts. First, there was the development of the inward FDI (IFDI) pattern aimed to boost the domestic economy of China and its capability for exporting manufactured goods. This began slowly with the door not so much ‘open’ as ‘no longer closed’, after the first 20 years of China’s Communist Party regime, Foster (2014). Then, from around the mid-1990s, the process gained momentum as Table 1 illustrates. Another 10 years on, around 2005, one can begin to see the significant growth of China’s outward FDI (OFDI). China had become a wealthier country with serious earnings which it wished to invest overseas, broadly speaking for the usual range of reasons detailed in the location aspect of Dunning’s OLI model: market seeking, efficiency seeking and resource seeking, see e.g. Dunning (2001).

**Table 1:** Inward and Outward FDI Flows for China 1990–2019 (in $bn) plus GDP. Source: UNCTAD Statistics, http://unctadstat.unctad.org/ (WIR 2020 Annex for FDI)

Year	1990	1995	2000	2005	2010	2011	2012
China IFDI	3.5	37.5	40.8	72.4	114.7	124.0	121.1
China OFDI	0.8	2.0	0.9	12.3	68.8	74.7	87.8
China GDP	404.5	757.0	1192.1	2287.2	5949.8	7314.1	8532.2

Year	2013	2014	2015	2016	2017	2018	2019
China IFDI	123.9	128.5	135.6	133.7	136.3	138.3	141.2
China OFDI	107.8	123.1	145.7	196.2	158.3	143.0	136.9
China GDP	9570.4	10,475.7	11,061.6	11,233.3	12,310.4	13,894.8	14,392.9

Table 1 below sets out the basic pattern of IFDI and its partner OFDI since 1990. We can see the cross-over point where OFDI matches or in some years exceeds IFDI at around 2015.

The data shown in Table 1 represent actual monies invested by Chinese firms in OFDI projects, whereas those shown in Table 2 are the total values of the projects into which the PRC monies invested were sunk. Scrutiny of the two tables shows that the numbers in Table 2 are from 1.4 to 2.2 times as great as those in Table 1. The AEI- china-global-investment-tracker source data show that the PRC participants, when not wholly funding projects, invested in stakes worth from as little as 1, 3 and 4% to 96–99% of project values. Again both data series show a growth from 2005 to 2017 by a factor of 10 or more. It is now a fact that China is one of the biggest sources of FDI in the world economy. The other point which becomes apparent, and is very interesting, to emerge from this data set is the wide dispersion of the hosts for China’s OFDI. It ranges from the developed economies of North America and Europe, through countries at varying stages of development across East and Southeast Asia to South Asia, Africa and South America.

**Table 2:** Total Value of PRC ‘OFDI invested’ projects 2005–2017, $bn. Source: AEI data: http://www.aei.org/china-global-investment-tracker

Year	2005	2010	2011	2012	2013	2014	2015	2016	2017
OFDI Projects	20.5	127.8	122.9	141.5	153.2	178.4	206.2	276.6	270.6

**Fig. 1:**
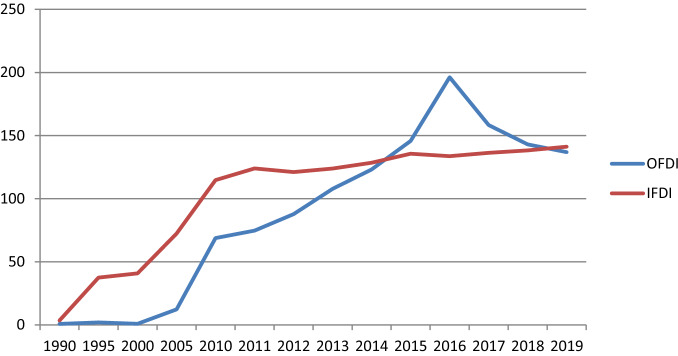
China OFDI v IFDI-$bn, UNCTAD Data

Whichever data set one considers, a key point to emerge is that it is really only since 2005 that China’s OFDI has begun to grow aggressively. Figure 1 shows this very well using the data from Table 1. The other notable fact observable in Fig. 1 is the turning point in the graph of OFDI at 2016. Beyond that OFDI has fallen each year; indeed, it fell by just over 30% over the next three years. We discuss possible reasons for this trend (or is it merely a hiccup?) in the final, discussion section of the paper. IFDI meanwhile, having hit a local maximum in 2011, has stayed fairly stable since then, increasing by a modest 14% (or an average 1.3% per annum).

The main aim of this paper is: to examine the key reasons for the locations of and the reasons for China’s OFDI as it booms and becomes a major OFDI player in the global economy; and at the end to assess the existence of possible risks to such a boom continuing. As noted above, there was a turning point in the OFDI graph around 2016. Part two of the objective concerns the question of why such a stall may have occurred and whether the pattern will be a sustained, at least in the short to medium term. The paper is novel in that it seeks to pull together three different elements of China’s OFDI story: a scrutiny of the pattern of aggregate flows; an understanding of the range of differing reasons for the making of and the placing of OFDIs; and, a scrutiny of potential, largely political, risks to the pattern of growth seen in the twenty-first century. There is a simple logic to the three parts of the overall aim. Consideration of the aggregate data flows establishes an emerging trend; the second element concerns factors underlying the trend; and then, since the upward trend faltered, there is the issue of possible inhibitors to its continuation, one of which may well be political risk.

As a starting point to the search for the answer to the first aim, consider the major reasons for companies making FDIs. The underlying theoretical reasons for making an FDI, and hence by extension an OFDI, are relatively few and are well known, see for example Dunning (2001), Rugman & Li (2007) and Williamson (2010). They are: market extension for the firm in question (often a motivation for firms with surplus capital available to invest); efficiency seeking (e.g. minimising production costs by using cheaper labour or establishing regional production pods to minimise shipping supply costs); and resource seeking (gaining access to scarce raw materials for one’s own firm or other firms in one’s domestic economy). The efficiency motivation will cover or include control of the raw material supply chain. The other factor which may be said to exist is a political motivation, which is germane to the China context essentially because of the significant presence of PRC government owned, or part-owned, companies (such as CNOOC or CITIC) whose motives are not necessarily fully rooted in micro-economic factors. Those political motives will include government desire to buy up resources (e.g. minerals in Africa) allied with control; desire to access and to feed back intellectual property from acquired overseas companies; and desire to maintain stocks of key, overseas currencies by owning revenue generating overseas assets. While some authors may claim to see other reasons for deciding to make FDIs they are in fact covered by those we have just enumerated, we should argue. There have been papers offering general overviews of the policy issues driving Chinese OFDI, such as Buckley (2019) and Davies (2012); we examine them further in the next section, which we feel is their natural place.

The only other issue not so far mentioned is the acquire or develop issue, i.e. whether to buy a ready-made business from another firm or whether to build one’s own, new subsidiary (as an FDI investment necessarily is) from scratch, so –called greenfield development. That, however, is not our real concern here: for, that choice is one of implementational strategy rather than developmental or growth strategy, see for example Demirbag et al. (2008).

The main body of the paper from here, in the next section, takes the form of a review of available secondary data, both numerical and published articles. As we explain in the third section, ideally one would like to underpin the arguments as to reasons for OFDIs with up-to-date, primary data but this proved infeasible; hence the reliance on secondary data. Apart from anything else, such primary data would facilitate an understanding of decision making at the level of the individual enterprise, whereas a good proportion of the prior published work relies on inferences as to firms’ decision making rationales from analyses of panel data. The final section is a discussion and conclusion, in which we not only present the answers to the first part of the objective but develop an argument as to the possible reasons for the stall in the growth pattern of OFDI.

The research is based primarily on secondary data, the numerical data already referred to above, plus a large number of published articles, most of which come in the two parts of the next section. As such the paper is essentially a review of the literature from which patterns and issues are discerned, a key issue being potential risks, from a Chinese perspective, to future progress. Our initial intention was to supplement this review with a modest sample of primary data, collected from officers of PRC companies, which have engaged in OFDI activity, by means of a semi-structured questionnaire. As we explain in the third section, we were not able to fulfil this ambition essentially because of the heightened tensions within China in recent years. Such tensions have made people wary of affording help to researchers in relation to a topic with ‘political aspects’.

## The literature on OFDI from China

### A picture of the development of OFDI from China over the past 25 years

The progression of value of OFDI is as outlined in the Introduction but what have been the motivations identified thus far in the story?

In an early examination of OFDI, Tseng (1997) looked at first time overseas investors. He found that, based on the data he assessed some 20 years ago, they were rather naïve in some ways. Their pre-investment screening visits to aid project assessment were often poorly organised and, the investment once made, they tended to lack the management skills necessary to adequately oversee them. Projects were often low technology, except where acquisition of a technology was the motive for investment. They tended to be greenfield projects of modest scale; often used a JV model; and viewed ethnic and cultural ties to be important. Another reason for early investment in some developed economies such as the USA and the UK pre-2000 was as a learning mechanism in technical industries such as the finance and insurance industries, see for example Zhang (2003)

Given the profile suggested by Tseng, it would have been hard to see Africa, say, as a likely host for PRC OFDI at the time he was writing but twenty years on we find that Africa is indeed a place which hosts such investment as the data underpinning the summary data in Table 2, which were compiled by The American Enterprise Institute and The Heritage Foundation (AEI for short), illustrate, AEI China-global-investment-tracker (2017). Over the period 2005–2017, Chinese entities invested not only in the more stable countries of the continent but also in some of its more unstable and risky countries such as DR Congo, Ethiopia and Guinea, a point reinforced by Ramasamy et al. (2012), this being particularly true for PRC-state invested entities.

Scanning down the spreadsheets of the AEI data base for 2005–2017, AEI China-global-investment-tracker (2017), some simple patterns emerge. Developed economies such as the USA and Europe experienced the acquisition of property and other established assets by China, as well as investment in new industrial projects. Hence, one of the reasons for investing in the developed economies would appear to be simply putting surplus funds to work to make a return, with the added benefit of the earnings being in foreign currency be it US$, Euros or £Stg. In less developed areas such as Africa and West Asia, construction projects and resource extractive projects featured strongly—the construction covered both real estate and investment infrastructure projects. This latter pattern suggests investment aimed at least in part, if not wholly, at providing the fuels and minerals required to support China’s manufacturing base with the infrastructure being a support function for the former type of investments. The only real peculiarity of this data base is the geographically odd regional placement of some countries. For example, one finds Morocco and Sudan ‘placed’ in the Arab Middle East and the Russian Federation in West Asia.

**Fig. 2:**
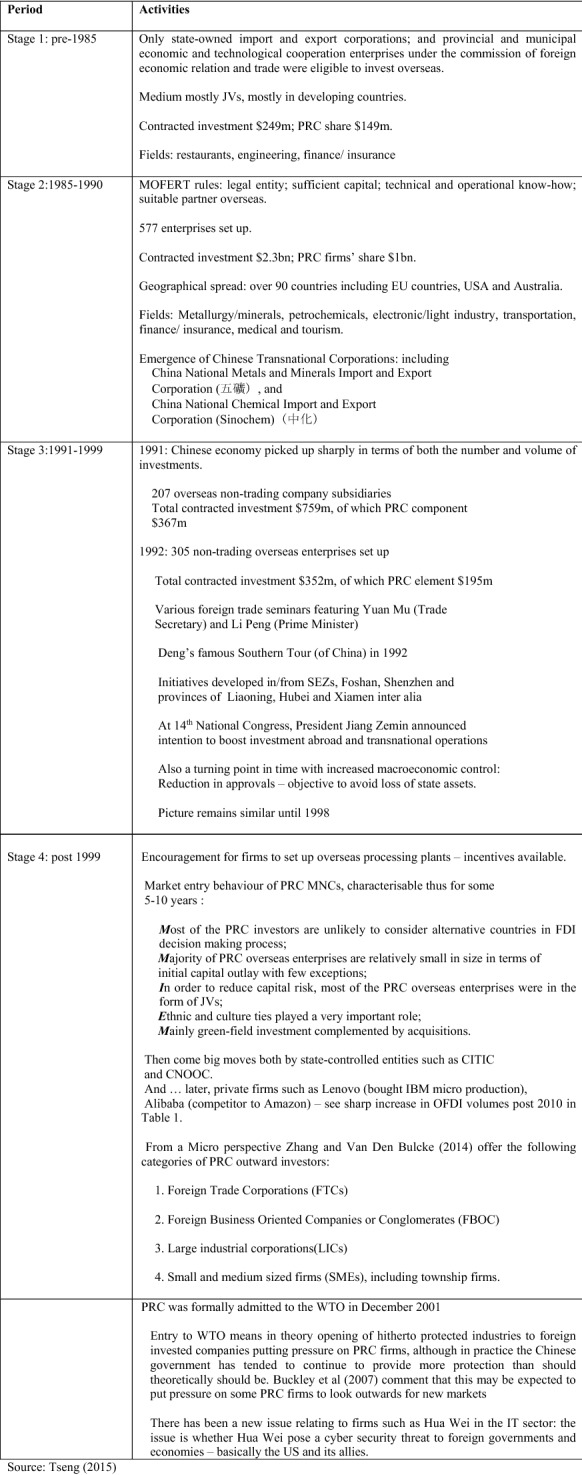
Stages of OFDI 1980 to the 2000s. Source: Tseng (2015)

In the past 15 years or so, there have been an increasing number of academic studies seeking to examine China’s OFDI. Early in that time period, Child and Rodrigues (2005), made the important point that not only were standard market seeking and physical resource accession objectives in play but Chinese, manufacturing MNCs were also seeking to acquire sophisticated technology or advanced manufacturing know-how by acquiring foreign companies or their subsidiaries. Morck et al. (2008) reported that then recent economic data suggested that the infant stage of China’s OFDI was biased towards tax havens and Southeast Asian countries with state-controlled enterprises, with government sanctioned monopoly status, prominent among the investors. Staying with the notion of government involvement in OFDI, Wang et al. (2012) found government support to be an important factor, while Cui and Jiang (2012) noted that the presence of SOEs as outward investors made joint ownership a more likely vehicle for investments. An important benefit for SOEs is identified by Sauvant and Chen (2014): namely, that China’s regulatory regime, while still somewhat cumbersome, as they wrote, in effect offered preferential treatment to SOEs. As noted a little later in Fig. 2, SOEs were particularly prominent in the picture when the PRC first made the decision to consider OFDIs in the 1980s; they led the charge so to speak, at the government’s behest.

In one sense, it is obvious that China’s SOEs will necessarily be best positioned to enact the policy objectives of the state precisely because they are owned, or largely owned, by that very state—so for example, scrutiny of the AEI database, AEI China-global-investment-tracker (2017), shows that CNPC, a state-owned energy company, could be found accessing oil and gas assets in quite a large number of countries, thereby fulfilling the PRC’s desire to secure energy resources needed to sustain its economic growth. Paradoxically then, an econometric study by Huang et al. (2017) claimed to find that central SOEs (as distinct from what they termed ‘local’ SOEs) were less likely to engage in OFDI. They suggest, however, that the negative effect of the percentage of shares owned by the state on those SOE’s OFDI will be mitigated by institutional developments and competition intensity. There is also the point that some SOEs are investment holding companies set up precisely for the purpose of building asset portfolios, for example CNOOC or CITIC, already referred to for that role in the Introduction. Their propensity for OFDI may quite naturally differ from that of a company whose initial purpose has been to fulfil a domestic production role.

Continuing with the idea of home country influence, Peng (2012) commented that, while much of Chinese MNC behaviour is similar to that of other MNCs, a differentiating feature is the role of the home, PRC government as an institutional force. This must presumably be strongly related to the presence of many SOEs in the set of outward investing Chinese MNCs. Peng noted two other factors which are important constraints to outward investing Chinese MNCs. These are: the challenge of making investments without having superior technological and managerial resources; and, their tendency to quickly adopt acquisition as the main mode of entry. Put simply investing without superior resources or acquisition experience behind them raises the inherent risks.

Slightly more recently, Huang and Renyong (2014) looked at issues specifically related to privately owned enterprises (POEs). They found that POEs felt themselves pushed towards market and strategic asset seeking OFDIs by the unfavourable institutional environment which they face in China. When making such OFDIs they tended to cluster with business partners or domestic peers. It remains to be seen whether, given time, recent PRC policy development may mitigate that perceived disadvantage.

Davies (2012) provided an overview of how China’s OFDI had continued to grow over the period 2008–2012, despite the then recent global crisis. The PRC Five Year Plan with effect from 2011, strengthened the commitment to promote the existing, “going global” policy. He records that, while much of the country’s OFDI continued to go into tertiary and primary sectors, there were signs of gradual sectoral diversification. He also highlighted the role of Hong Kong and the Caribbean as venues through which China’s OFDI is intermediated. Thus when the British Virgin Islands (BVI) appears in the data as the destination for a major investment by an MNC it does not mean that any primary or secondary industrial activity is taking place in the BVI: simply put the investment is routed via the BVI for tax efficiency reasons, see also Morck et al (2008) above. Again, Ramasamy et al (2012) make a similar point based on their empirical data. Regarding this type of use of tax havens/offshore financial centres (THOFCs), Sutherland et al. (2019) note that there is an important side effect of this type of behaviour when attempting econometric modelling of reasons for OFDIs arriving in their target countries. If the data from databases used in models are not corrected in some way to allow for the THOFCs being ‘false destinations’, the result will be unrealistic models in terms of the underlying reasons for outward investment. In turn, recommendations relying unduly on such models may be ill-advised.

In their study of investments by large state-owned Chinese firms in Sub-Saharan Africa’s resource and infrastructure sectors, Kaplinsky and Morris (2009) found that such investments were closely bundled with aid and trade. As they put it, the PRC firms access resources for their domestic economy and build political leverage through the enactment of infrastructure projects. Moreover, as we noted earlier, investments of this kind continue to feature in Africa, as shown in the AEI data base, AEI China-global-investment-tracker (2017). Another interesting point, albeit derived from pre-2001 data, concerning the character of China’s OFDI is made by Buckley et al. (2007). They found that Chinese OFDI was associated with high levels of political risk in host countries: this resonates with our earlier point concerning investments in unstable African countries, catalogued in the AEI data base. More recently, Donou-Adonsou & Lim (2018) examined the effect of Chinese OFDI on economic performance in Africa. Broadly speaking, they suggest that Chinese FDI improved income in Africa and that there is some evidence that Chinese investment tends to crowd out U.S. investment in Africa, an impact not experienced by French investment they suggest.

In a study examining data of Chinese OFDI to 75 host countries over the period 1994–2005, Zhang and Roelfsema (2014) found certain clear trends emerging, albeit the big surge in OFDI was only just beginning by 2005. In particular, they reported increased market commitment and external resource seeking behaviour (both natural resources and strategic assets), all of which fits with other findings. In a much more focussed study of investments into the USA over the period 2003–2011, Anderson and Sutherland (2015), concluded that their analysis broadly supported the view that acquisitions were the primary mode of effecting strategic asset seeking in a developed market such as the USA. Whether they would regard the kind of purchases Alibaba make as acquiring strategic assets, their purchases are there in the record (six deals in 2013–16). CIC’s financial sector purchases meanwhile are certainly strategic and some of the deals are large involving seven figures in US$, see AEI China-global-investment-tracker (2017).

Looking specifically at provincial OFDI from China, Chen (2015) concluded that the main motives for China’s provincial firms to invest abroad are market seeking and efficiency seeking. They examined data over the period 2003–2012, with OFDI from provincial firms going up by a multiple of about 8 between 2008 and 2012, after a more muted growth in the preceding five years.

Tseng (2015) offered a useful snapshot of the unfolding of the Chinese OFDI picture from a macro-perspective, over the period 1980–2000 and beyond. In summary, he described the following patterns.

We look now at six very recently published papers to see what they may add to the picture. Liu et al. (2018) explored the link between cultural distance and OFDIs. Overall, their data suggested that composite cultural distance had a U-shaped relationship with Chinese OFDIs. But this picture was not uniform across the cultural dimensions. Intuitively, a ‘reversed-L’ shape relation might have been more to be expected.

Buckley (2019) presents a fairly general picture of China OFDI evolution. He says in his abstract (p6) that, “[there exists] evidence of coordination of Chinese OFDI but also context, conflict and independent decision making (in Chinese firms) play a role in the determination of direction, control and outcomes of the OFDIs.” Later in the paper, he says anecdotally that some large Chinese MNCs with deep pockets seem to acquire businesses without any obvious coherent plan. Put another way they are making themselves into geographically dispersed, diversified conglomerates. In western economies, such diversified groups are much less fashionable than they were 30 years ago but who is to say they may not yet come back into fashion, given time, [e.g. who now instinctively knows names such as Hanson (James of that ilk), BTR or knows that 30 years ago BAT were big players in Finance as well as tobacco?].

Papageorgiadis et al. (2019) examined the link, or otherwise, between intellectual property institutions in 23 European countries and flows of Chinese OFDI (COFDI). In line with the hypotheses they set up, they found that those European countries with strong IP institutions tended to attract higher levels of COFDI; and that the strength of European IP institutions has a U-shaped relationship with COFDI. This U-shaped curve is explained by the fact that some Chinese firms were attracted to the weaker IP institutions in former Eastern Bloc European firms, rather in the vein reported by Ramasamy et al (2012), see earlier. What these results suggest to us is that well run Chinese firms may have an appetite for a quality regulatory environment, perhaps not least for the certainty which such an environment offers.

Still focussing on Europe, more exactly the European Union (EU), Leung (2019) explored Chinese OFDIs into the EU energy market/s over the period 2005–2015. He reports that energy investments accounted for 31% of PRC investment into the EU in the stated period, enacted through 37 deals, more being post 2010 than prior thereto. Most interestingly, he further found that since 2011, there has been a change away from investments in ‘traditional’ carbon based energy firms to greener types of energy (including nuclear power). It may seem to some to be invidious to criticise him for describing nuclear power as a ‘clean energy’, since it is not an uncommon practice but we beg to differ. No-one has yet found a satisfactory solution to the problem of dealing with spent fuel rods from nuclear plants which by any reasonable standards are pretty ‘dirty’. They don’t give off carbon emissions—CO_2_ or CO—but they are dangerous. ‘Safe storage’ pro tem is not a long-term solution, always provided man has not made planet Earth uninhabitable before such a solution can be found.

Meanwhile, investments by China in the EU in truly clean energy sources such as hydro-power, solar-power and wind-power are good news both for any EU countries so invested and for China itself. China itself because there will be inevitable learning from its investments in the EU, or indeed elsewhere, which should help it domestically as it tries to clean up its own pollution problems. This last point is essentially that made by Zhou et al. (2019a, b). They found that China’s OFDI does indeed bring with it green spillover effects back in China, not solely in the energy sector. They examined a data set disaggregated to provincial level. While as noted green spillover benefits were found to occur, they were not evenly distributed across the set of provinces. This variability across provinces was found to be related to, amongst other things, education levels and investment critical mass. However, as with Leung’s work, it seems only sensible to applaud and encourage any activities which improve greening in the PRC economy and improve efficiency. Small steps can lead to bigger ones given the will to act.

The final one of the six papers mentioned is that by Zhou et al. (2019a)—a different Zhou. They found that domestic innovation performance (DIP) was positively related with COFDI into developed countries, in other words there is a learning and feedback loop in play. On the other hand, they found a tendency to a negative relationship between DIP and COFDI in transitional and emerging nations. This is not very surprising: as we noted earlier Chinese investment in some emerging African counties is targeted at acquiring minerals for domestic Chinese use. Other investments, in transport and power plants may be largely self-serving in their objectives, i.e. facilitating the primary goal of mineral acquisition.

Most of the work to which we have referred above concerns the actions and intentions of the individual firm, whatever the character of its ownership in the PRC may be. In a recent Columbia FDI Perspective, Zhang (2019) reflects on the trend in China’s bilateral investment treaties. He opines (p1) that in future, “China can be expected to emphasise outward FDI protection more, especially in implementing the Belt and Road initiative.” In addition to bilateral investment treaties, another factor which may affect China’s OFDI is its revised regulations for OFDI and allied guidance to Chinese banks on associated lending, see Baker McKenzie (2018). Indeed, Baker McKenzie argue that these changes have been a major factor in the reduction of China’s OFDI since its 2016 peak.

A new highlight come 2020 is what may be called the ‘Huawei saga’. The issue at hand is whether use of Huawei products in European, North American or Australasian communications systems poses a threat to the national security of countries in those regions. Huawei say ‘no’ and deny that they act, in part, as proxy agents for the PRC. The CIA and British and Australian intelligence services are less sanguine on the issue. The Chinese Communist Party (CCP) and PRC government officials meanwhile express outrage at the mere suggestion that they would use Huawei kit in new, 5G communication systems to spy on the west, be it political spying or industrial espionage.

The title of one recent paper by Hao et al. (2020) poses an interesting question. ‘Does outward foreign direct investment (OFDI) affect the home country’s environmental quality?’ Their conclusions are mixed in terms of positivity or negativity of impact. On the positive side, they suggest that a reverse technology spillover effect of OFDI improved the domestic technology level and the domestic industrial structure, with a consequent positive impact on (or reduction in) domestic pollution. On the debit side, their analysis suggested that China’s OFDI has increased domestic environmental pollution due to allied boosts in economic scale.

A recent BBC news report, (BBC 2021) stated that in 2020 only 20 investments were made by China into Australia (down from a peak of 111 in 2016), worth about $800 m. This was a second year of falls with a 47% fall in 2019, when investments were worth $1.57bn. In 2020, it is reported that the only three significant areas for investment by China were real estate ($357 m), mining ($321 m) and manufacturing ($119 m). The report suggests that a major cause of the recent sharp decline in OFDI by the PRC into Australia was the Australian government’s call last April for a rigorous investigation into the source/s of the COVID-19 pandemic—a key element of the concern was whether China had failed to be transparent about the problem. Before that and also during 2020, Australia has taken a firm line with Beijing on other issues such as the repression of the Uighers in western China and freedom of navigation rights in the South China Seas. All of these issues have helped to sour diplomatic relations between the two countries. Although FDI flows faltered globally in 2020, the falls in Chinese OFDI to Australia were still well in excess of that global trend.

Not only has there been a fall in Chinese OFDI into Australia but tariff and non-tariff barriers to Australian exports have been put in place by Beijing. These restrictions have affected trade in a range of product areas including coal, barley, wine, beef and lobsters. In the case of wine some products have had tariffs in excess of 200% imposed by China and in the case of coal events have seen laden, coal-carrying vessels stranded off the Chinese coast, unable to land their cargoes.

A rather more long-standing example of politics affecting China’s OFDI is what may be called ‘the Taiwan issue’. For example, Tuman and Shirali (2017, p 154) found that, “[their] study adds to the prior literature by demonstrating empirically that Chinese FDI flows are negatively associated with recipients who maintain diplomatic recognition of Taiwan.” See also Eyal (2021), who look rather at the European position in regard to China's perceived threatening attitude with possible consequences for trade.

Finally on this aspect of possible political influences in relation to Chinese FDI, Lu and Biglaiser (2020) report interesting elements of such FDI into the USA. Their econometric modelling suggested that such FDIs were more likely to occur in Republican governed states, particularly in the case of greenfield investments—a conclusion which strikes us as intuitively surprising. They also found that US national security concerns were having an impact in hi-tech sectors. Viewed through another, more economically rooted lens, this paper can be seen as evidence of differential patterns of Chinese OFDI arrival across the states of the USA. Another example of differential patterns of sub-national arrivals of Chinese OFDIs within a country is reported in Mexico by Tuman and Erlingsson (2020). They concluded from analysis of a sample for the period 2004–2014 that market size, education levels, and partisan control in state government were influencing factors on inbound FDI decisions, as was the availability of deep-water ports in particular states.

### Does IFDI have an effect on OFDI or not?

In the previous sub-section, we focussed on literature examining firms’ investment objectives and hoped for benefits from China’s OFDI. Here we look, more briefly, at whether evidence has been found to suggest any impact of IFDI on OFDI in China.

Some authors have suggested that firms in developing countries (of which China is one) where strong inward internationalisation is found tend to show more intent to internationalise outwards, see e.g. Luo et al. (2011) and Luo and Wang (2012). On the other hand, it has been argued that, where positive spillover effects from IFDI occur in a given economy, firms’ tendencies to make OFDIs may be reduced precisely because of those ‘at home’ benefits, e.g. Li et al. (2012). However, not all studies looking for potential spillover benefits from IFDI find such benefits, see e.g. Gorg and Greenaway (2004).

Liang et al. (2012) go so far as to suggest that Chinese firms who do not have any foreign investment may be likely to engage very aggressively in OFDI to try to off-set a perceived disadvantage in their home market.

Ma et al. (2015) looked at this question by means of a case study based examination of the automotive industry in China. They made in-depth examinations of three firms, Shanghai Automotive Industry Corporation (SAIC), Nanjing Automobile Corporation (NAC) and Zhejiang Geely Holding Group (ZGG), all of whom had made OFDI moves. They looked at a large amount of published information about the firms and, it seems, were afforded an interview of at least an hour at each firm. Obviously generalisation of any conclusions would be difficult given a sample of three. Nevertheless, the key result which they reported themselves to have found was (p. 289): “We propose that IJVs may have a negative effect on both firms’ motivation for venturing abroad and the degree and speed of internationalization of Chinese automakers. In addition [they] suggest that Chinese automakers without IJVs may adopt dual strategic motives for internationalizing.” Those two features were: making rapid and risky moves to try to ‘catch up’; and using new capabilities learnt abroad as a springboard to grow their position within the domestic China market.

## The primary evidence issue and consequent insights

When we began thinking about this paper, one of the planned, and obvious, stages was to be the collection of at least a modicum of new, primary data: we hoped to gather fresh data from PRC firms exploring the questions posed in the Introduction. The problem which arose was that the global context in which China exists and hence within which PRC firms may seek to make OFDIs has become at the very least ‘rather tricky’. This is because of disputes between China and possible sink countries and also because of the PRC government’s excessive sensitivity to any slights, real or imagined, as referenced below in this section. Operating in a distinctly Chinese fashion, we sought to exploit our business networks to try to gain access to deliver responses to a semi-structured questionnaire. What we found was that it was difficult to get the access which we probably would have achieved 10 years ago. Network contacts were friendly but very cautious. Had implementation of the questionnaire proved feasible, it would have covered inter alia issues such as a company’s: scale of OFDI; hoped for aims of the OFDI; location of OFDI; reasons for choice of location; sectoral interests of the OFDI; ownership type of the company (e.g. state or partially state owned, or private); and, influence of PRC government policies on the attempted OFDI. It is our view that such data, directly achieved had it been possible, would be much more reliable than the implied answers to such questions as inferred by the econometric models from which inferences are often drawn. To slightly misquote E.F. Schumacher, the renowned, heterodox economist (but entirely in the spirit of the original), ‘an ounce of primary information may be worth a ton of theoretically inferred information’, BBC (2016).

One woman, known to us for over 20 years, wrote that she felt many of the people she and her businessman husband knew might feel that making overt, public comment could be ‘dangerous’ for them, in the world of 2021. She did not elaborate why such might be so but China’s combination of a more hostile approach recently towards a range of foreign countries on various issues and internal crackdowns by the Chinese government, under Mr Xi, make for a somewhat tense internal setting in the PRC. China has had troubled relations with: the UK over Hong Kong and the Xinjiang Uigher issue; the USA over US-PRC trade, Xinjiang and Taiwan’s status (see the earlier reference to Tuman and Shirali 2017.); Australia for their support of the Uighers inter alia; and regional neighbours round the South China sea (the Philippines, Vietnam, Indonesia, Malaysia) over the so-called 7-stroke map whereby China seeks to lay claim to nearly all of that sea area. The last named of these ‘pressure points’ has two aspects: construction of offshore military bases, adapting attols/small islands by land-build for such bases; and, making attempted claims to undersea mineral deposits in waters to which other countries believe themselves to have registered, and recognised, international claim.

When Xi first came to power in 2013, the crackdown he announced on corruption in China was seen by many observers as a step in the right direction. Others, however, were more sceptical. Of course, they argued, corruption was a well-known problem in China which required attention but might it be that the Xi government was, at least in part, clearing out political opposition under the guise of the corruption crackdown? (see for example: Economy 2014; McGregor 2019). It has even been suggested (see for example Zhu and Zhang 2017), that getting rid of political opponents was the prime reason for Xi’s anti-corruption campaign. In this kind of setting, it might be time to keep one’s head down.

Anyone who thinks this is fanciful thinking might care to look at the case of the businessman Jimmy Lai in Hong Kong who has been arrested and charged on suspicion of, inter alia, sedition under the new PRC enacted laws imposed on Hong Kong, laws whose compatibility with articles 4, 18 and 23 of the Basic Law (1990) are highly debatable. In the second week of March 2021, the People’s Congress, meeting in Beijing, rubber stamped (by a unanimous vote!) new laws concerning who may stand as candidates for Hong Kong’s Legislative Council (the colony/SAR’s local seat of government, commonly called Legco). The new rules basically say that you have to be overtly prepared to follow Beijing’s policy line in order to sit in Legco, or to be ‘patriotic’ as they style it. This again seems to be evidently contrary to the Basic Law of Hong Kong. However, any airings of such reasonable inferences are summarily denounced by PRC or Hong Kong Government spokes(wo)men, see for example the recent press report by Lam (2021).

As we further explain in the Discussion which follows, the kind of political tensions mentioned here, internal and external to China, are potential inhibitors to the future development of China’s OFDI.

## Discussion and conclusion

China’s position as one of the world’s major outward foreign direct investors is now clearly established as was illustrated in Fig. 1. Prior to about 2005, Chinese OFDI was very minor for a country with an economy as big as theirs and, when first allowed, OFDI was restricted largely to SOEs and firms linked to other tiers of the political hierarchy such as provinces (see Fig. 2, Stage 1). Now, China is one of the major sources of FDI across the globe. The PRC’s reasons, and those of some key ‘new capitalists’ such as Jack Ma (of Alibaba fame), for promoting outward FDI from China have been various. State owned or supported businesses have invested strategically in industries such as mining, alternative energy generating equipment and construction materials. Put simply China needs oil, coal and alternative energy sources to keep its industrial heart throbbing. It needs ores and metals for the production of the likes of steel and aluminium on the one hand and as input materials for industries such as computing and other high-tech kit makers. When investing in mining in countries in Africa and South America, China has facilitated its access to the mined products it seeks by helping to improve infrastructure at the location of the OFDI. It has also invested in Australian mining but there have been recent ‘difficulties’ in Australia as noted earlier.

In other industries, they have simply sought to invest in businesses which they believe or hope will deliver economic rent: this has tended to be the focus of what we referred to before as the ‘new capitalists’. Such industries may include real estate, electronic engineering and building supplies (e.g. cement). In the S.E. Asian area, Chinese state supported businesses have invested in this second category of business type to take advantage of low-cost labour and to access the ambient market of the consequent production facilities.

Another benefit from OFDI which may occur across a range of business segments is the acquisition of technology or systems procedures or understanding to enable improvements to institutional structures for reverse application in China. In some cases, such reverse engineering is a direct objective of the OFDI, in others, it is more in the nature of an unexpected side benefit from the OFDI. Either way, it is an example of one kind of spillover effect, several of which were noted in the main data review section.

Telecommunications are another important area for investment and, up to about the end of 2018, China may have thought it was going along quite nicely with Huawei busily supporting 4G and then 5G telecoms installations and end-user kit. However, that has all gone rather off track from the PRC perspective once major western economies began to doubt the wisdom of allowing a company such as Huawei to be involved in the development of their 5G networks because of fears relating to cyber-espionage—of both the industrial and the political type. The problem for China is of course that they have form for such espionage developed over a substantial period. Reliable sources reckon that the PLA have long had a major computer espionage set-up, based in Greater Shanghai, so why not go one step further and pop malign access devices into new systems with which one is engaged? This is not to suggest that China is alone in covert (and illegal) monitoring of foreign entities. Of course, Russia and the USA do it too but, from a European perspective say, it may be thought unlikely that the USA will seek to deploy its cyber spying capabilities in order to do active harm to its NATO partners. The fear is that China and Russia will have no such qualms. Were that to be a misapprehension then why has China reacted so badly to Australia, as outlined earlier, and why does it build military bases on little islands in the South China Seas in waters which according to the UN ratified international map of the area are the waters of other countries in the region (see for example, Storey 2021)?

One possible conclusion from the foregoing is that China may be at risk of jeopardising its own business development chances, by overplaying its hand in S.E. Asia and by engaging in global spying elsewhere. China’s achievements in first building up and modernising its domestic economy and now developing itself further by overseas investment have been phenomenal by any standards. Why risk spoiling it all by inappropriate, hostile behaviour? Might China be advised to ‘look North’ and see how Russia (and before that the Soviet Union) managed to first build a position of major strength then sink back to a position as, at the very most, third string player in the geopolitical power game. Moreover, the Indian economy is growing and with it will come increased leverage. Might they soon be ready to take over if anyone slips and leaves a way through to the top?

In this context, the so-called Belt and Road Initiative (BRI) (for a detailed overview see OECD 2018) can be seen as a kind of touchstone: is it a positive move for both China and the countries in which they invest as part of the BRI programme or is it a thinly veiled power grab with potential ecological downside risks? There are certainly some who see it as the former, see Business Standard (2019a, b), and others who have identified fears regarding ecological issues at and beyond China’s borders, especially in Eurasia, see e.g. Tracy et al. (2017). They note potential pollution and bio-hazard migration as risks. In political terms, Friedberg (2020) goes so far as to suggest that China’s underlying intent in pushing the BRI is to establish itself as the self-proclaimed leader of the global South, achieving its aims by a mixture of authoritarian politics and quasi-market Economics. Nordin and Weissmann (2018) suggested that the Trump administration in the USA was unwittingly facilitating the PRC’s global economic momentum by itself adopting what they termed a protectionist stance. But, as we noted above, others are now beginning to see an alternative picture whereby the PRC’s ambition lacks ethical intent even in economic terms. Moreover, it is seen to be behaving in an overtly hostile and dangerous manner in the E./S.E. Asian region through military actions, see for example Storey (2021) and Yong (2021).

Another factor negatively impacting the trend of OFDI from China has been the change in their own regulations, as we explained in the second section.

In the short section on the interaction between IFDI and OFDI, we reported evidence from studies suggesting that IFDI–OFDI interactions can be both positive and negative in form. Elsewhere it has been reported that since becoming one of, if not always *the* top, sinks for FDI, China has, perhaps paradoxically, become a more hostile environment in which to operate for foreign MNCs, see for example Foster (2015), Foster & Tseng (2017). This again might be seen to be a misjudgement by the Beijing government, because the choice between a genuine level playing field or a hostile environment for foreign investors lies in the gift of the host government. If Beijing abuses its powers—whether in cyber-malfeasance, oppression of minorities, not adhering to WTO rules or military aggression in the East Asian arena—the rest of the world may simply decide that active steps are needed to ‘put them in their place’, as the saying goes. Were that to happen the Chinese economic miracle, including its new found OFDI position, might be put at risk. Indeed, there are some who think that a tipping point has already been reached: the west, be it the USA, Europe (the EU, the UK and others) or the G7, has had enough of the new bellicose China and is ready to oppose them, regarding the at times hysterical denunciations of any and all criticism as a symptom of weakness rather than strength, see Friedberg (2020), Associated Press (2021), Horton (2021).

Our paper underlines the point that the determinants of Chinese OFDI are several and varied, evolving from a state dominated activity to a more mixed economy, although SOEs remain important players in the COFDI mix. The objectives of such OFDIs are variously market seeking, efficiency seeking and resource seeking; in short, all of Dunning’s (2001) key rationales are represented. The last of these three is one which is particularly underwritten by the Chinese state by means of SOEs, with the mineral wealth of Africa a particular target. One of the key contributions of the paper is to highlight the potential risk to China’s OFDI (and perhaps also its IFDI) of its tendency to a rather bellicose approach to foreign affairs, a theme seldom addressed explicitly in the International Business literature. This may be seen as a rather domineering posture towards what are perceived by China to be ‘lesser’ countries where it chooses to invest as part of its Belt and Road Initiative on the one hand; and an intemperate attitude towards bigger, more developed nations should they dare to query the attitudes and demands of the Chinese Communist Party, overseen by its new ‘strong man’, Xi Jinping.

## Data Availability

I am the sole author. I do not believe a data availability statement is required since the paper relies on secondary sources.
